# Establishment of cell culture model and humanized mouse model of chronic hepatitis B virus infection

**DOI:** 10.1128/spectrum.02745-23

**Published:** 2023-11-29

**Authors:** Renyun Tian, Di Yang, Biaoming Xu, Rilin Deng, Binbin Xue, Luoling Wang, Huiyi Li, Qian Liu, Xiaohong Wang, Songqing Tang, Mengyu Wan, Hua Pei, Haizhen Zhu

**Affiliations:** 1 Institute of Pathogen Biology and Immunology, College of Biology, State Key Laboratory of Chemo/Biosensing and Chemometrics, Hunan University, Changsha, China; 2 Department of Pathogen Biology and Immunology, Department of Clinical Laboratory of the Second Affiliated Hospital, Key Laboratory of Tropical Translational Medicine of Ministry of Education,Institute of Pathogen Biology and Immunology,School of Basic Medicine and Life Science, The University of Hong Kong Joint Laboratory of Tropical Infectious Diseases, The Second Affiliated Hospital of Hainan Medical University, Hainan Medical University, Hainan, China; Shandong First Medical University, Huaiyin, Jinan, Shandong, China

**Keywords:** HBV persistence, cell culture model, animal model, kinetics of antigen, virion secretion

## Abstract

The absence of a hepatitis B virus (HBV) infection system has constrained the study of the viral life cycle and the development of antiviral drugs. We previously established a hepatocellular carcinoma cell line, HLCZ01, that can support the entire life cycle of HBV. To further optimize the model, we compared the kinetic process of HBV infection in HLCZ01 and HepG2-NTCP cells and found that HLCZ01 cells were more conducive to mimic the long-term stable infection of HBV in the natural state. By comparing the composition of the progeny virus and the differences in gene expression profiles between these two types of cells, we were able to explore the factors affecting the efficiency of virus infection. We built an experimental platform for human liver chimeric mice and established a standard surgical procedure. By transplanting HBV-infected HLCZ01 cells into mouse liver, which can be used instead of primary human hepatocytes to establish chimeric mice, this animal model can continuously produce viral particles. The establishment of these two models will facilitate the exploration of the mechanism of persistent HBV infection, the understanding of host‒virus interactions, and the determination of new targets for chronic hepatitis B therapy.

**IMPORTANCE:**

Approximately 257 million people worldwide have been infected with hepatitis B virus (HBV), and HBV infection can cause chronic hepatitis, cirrhosis, and even liver cancer. The lack of suitable and effective infection models has greatly limited research in HBV-related fields for a long time, and it is still not possible to discover a method to completely and effectively remove the HBV genome. We have constructed a hepatocellular carcinoma cell line, HLCZ01, that can support the complete life cycle of HBV. This model can mimic the long-term stable infection of HBV in the natural state and can replace primary human hepatocytes for the development of human liver chimeric mice. This model will be a powerful tool for research in the field of HBV.

## INTRODUCTION

Hepatitis B virus (HBV) causes chronic infection in approximately 257 million people worldwide, and such chronic infection can lead to chronic hepatitis, cirrhosis, and even liver cancer (data from WHO 2017). HBV infection, a global public health problem, is responsible for approximately 887,000 deaths annually. The lack of infection models has severely limited research in HBV-related fields, which further constrains drug development, and no method has been found to eradicate the HBV genome.

HBV belongs to hepatotropic DNA viruses, and its genome shows a partially relaxed loop structure (relaxed ciruclar DNA, rcDNA) with a length of approximately 3.2 kb (1). The specificity of the HBV receptor determines its tissue specificity of infection; the receptor conducts the initial interaction of the virus with heparan sulfate proteoglycans (2), enabling the virus to attach to the plasma membrane. Subsequently, the NTCP protein (3), which serves as its invasion receptor and is highly expressed only in hepatocytes, aids in internalization of the viral particles, and after fusion of the envelope with the cytoplasmic membrane, the capsid is released into the cytoplasm and transported to the nucleus. Near the nuclear pore, rcDNA is released into the nucleus, where it is converted to covalently closed circular (ccc) DNA. cccDNA serves as a transcriptional template for pregenomic RNA (pgRNA) and subgenomic RNA with the assurance of the persistence of HBV infection (4). pgRNA is packaged into the newly formed capsid along with HBV polymerase and undergoes reverse transcription to produce progeny virus. In addition to the ability to produce intact viral particles, the viral replication process creates a large number of subviral particles, called large spherical particles, small spherical particles, and tubular particles (5). These subviral particles may maintain persistent HBV infection by modulating the host immune microenvironment. Because of the tissue specificity of HBV infection and host specificity, the development of suitable cellular and animal models capable of supporting HBV replication remains difficult, and research in HBV-related fields is severely limited (6). Isolated primary human hepatocytes are known to support the complete life cycle of HBV, but the lack of sample sources, unpredictable genetic background, and harsh culture environments have limited their widespread use (7). The majority of hepatocellular carcinoma cell lines commonly used in the laboratory do not support the entire life cycle of HBV. HepG2/Huh7 cells support HBV replication but not invasion. The current cell models used for HBV life cycle studies are all genetically modified cell lines, except for HepaRG cells. HepaRG cells can support the life cycle of HBV by inducing differentiation over a long period of time, but they are not widely utilized because of their low infection efficiency, difficulty in inducing culture, and long experimental period (8). With the help of HepG2 cells transfected with the tetraploid HBV genome containing tetracycline resistance, cells of the stable cell line HepG2.2.15 were obtained using antibiotic screening, which can support HBV replication and the release infectious of viral particles but cannot support the invasion process of HBV (9). The HepG2-NTCP cell line obtained by overexpressing the HBV receptor NTCP in HepG2 cells can support the entire life cycle of HBV, and this type of cell line has been commonly used in recent years, but because it is a genetically modified cell line, it does not mimic the physiological process of HBV infection well (10). There remains a lack of cellular models for the dynamic observation of HBV long-term infection.

Compared to cellular models, animal models available for HBV research are also scarce (6). Commonly used experimental animals, such as mice and rabbits, do not support HBV infection, and although chimpanzees and tree shrews can support the life cycle of HBV, their further use is limited by difficult access to sources, complex genetic backgrounds, cumbersome manipulation, and numerous ethical restrictions. The construction of human liver chimeric mice by introducing human-derived primary hepatocytes into the liver of mice via a liver injury mouse model can support the entire life cycle of HBV (11). However, few sources of fresh human liver tissues, diverse genetic backgrounds, high construction costs, long experimental cycles, and difficult operation techniques have limited the application scope of human liver chimeric mice.

To develop an ideal HBV-infected cell culture system, we isolated a hepatocellular carcinoma cell line, named HLCZ01, which is morphologically closer to primary human hepatocytes, supports the complete life cycle of HBV (12), and has an intact innate immune response system that matches primary human hepatocytes (PHH) (13, 14). Based on this, we optimized and extended the system in this study. Comparing the model with existing common viral infection models, we found that this model not only supports the complete life cycle of HBV but also facilitates stable viral infection without significant loss of the viral genome. This model releases more infectious viral particles during infection and promotes the high expression of virus-related genes, and it can be used instead of primary human hepatocytes to construct chimeric mouse models for HBV life cycle studies and related drug development.

## RESULTS

### Morphological and biological characteristics comparison of hepatocellular carcinoma cell lines

**Fig 1:**
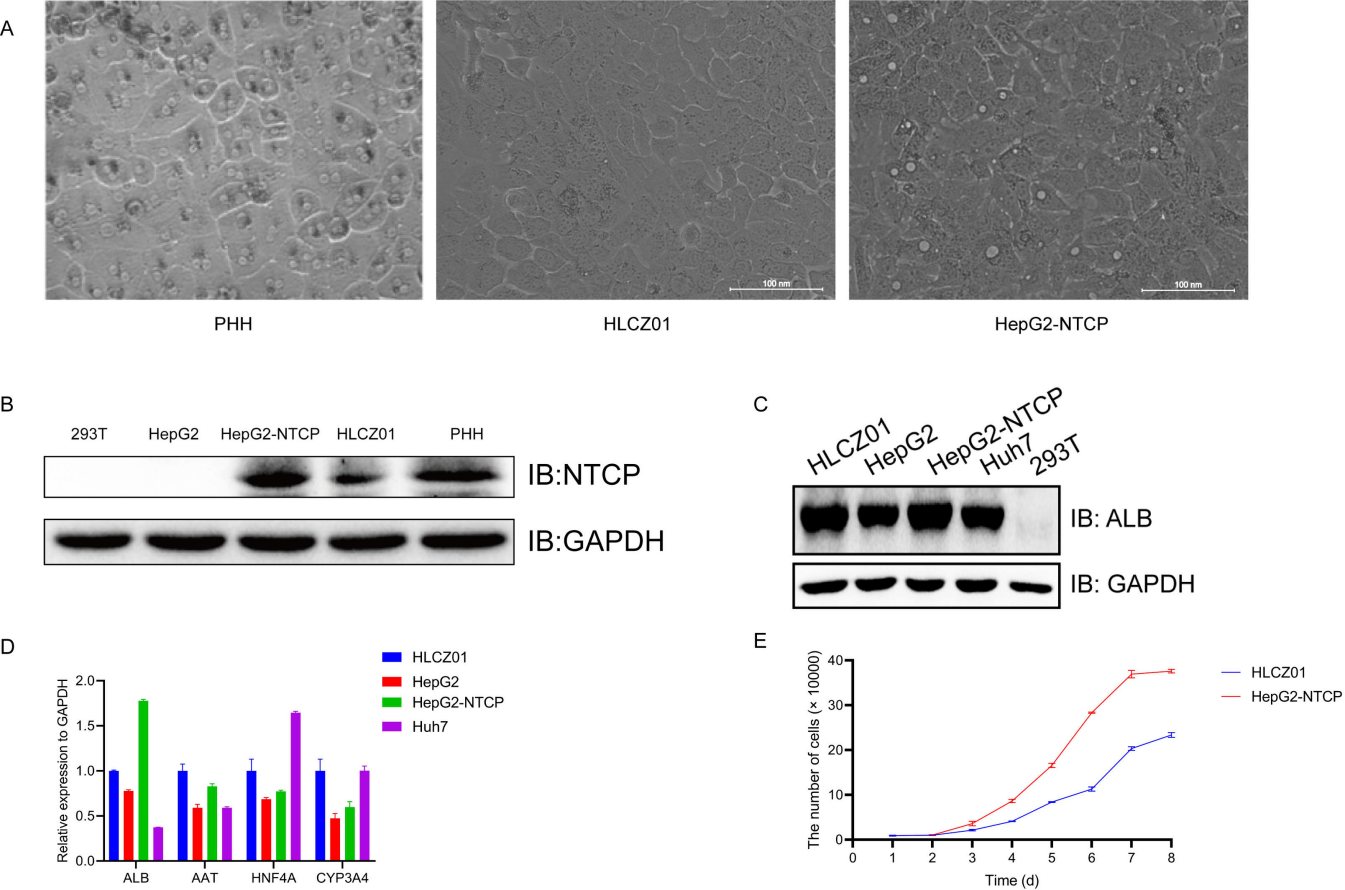
Morphological and biological characteristics comparison of the hepatocellular carcinoma cell lines HLCZ01 and HepG2-NTCP. (**A**) Morphology of HLCZ01 and HepG2-NTCP cells derived from hepatocellular carcinoma. (**B**) The overexpression effect of NTCP in HepG2-NTCP cells was detected by western blot. Protein was isolated from 293T, HepG2, HepG2-NTCP, HLCZ01, and PHH cells. (**C**) HLCZ01 and HepG2-NTCP cells express the liver-specific protein ALB. Protein was isolated from HLCZ01, HepG2-NTCP, Huh7, and 293T cells. ALB was detected by western blot. (**D**) Detection of human hepatocyte markers by real-time RT-PCR. Total cellular RNA was isolated from HLCZ01, Huh7, HepG2, and HepG2-NTCP cells. Human AAT, ALB, HNF4, and CYP3A4 mRNA were detected by real-time RT-PCR and normalized to GAPDH. (**E**) The growth curves of HLCZ01 and HepG2-NTCP cells were mapped by cell counting, and error bars represent SD from triplicate experiments.

In the following study, we found that HBV persisted stably (up to several months) in HBV cells by long-term passaging culture, indicating that this cell line supports long-term stable HBV infection, which is extremely rare in HBV-infected cell systems. To understand and utilize this model in depth, we compared this model with the commonly used HepG2-NTCP cell line. We screened for HepG2 cells overexpressing NTCP by culturing the cells with increasing concentrations of puromycin (up to 2 µg/mL) over 4 weeks (Fig. 1A). We compared the morphological characteristics and expression of major markers of hepatocytes in the two cell lines and found that HLCZ01 cells morphologically resembled primary human hepatocytes more closely (15), with a fuller morphology and clearer contours compared to the long shuttle-shaped structure of HepG2-NTCP cells (Fig. 1A). Western-blot assays showed the screening effect of HepG2-NTCP cells, as well as the expression of NTCP protein was confirmed in HLCZ01 cells by comparison (Fig. 1B). The major marker ALB was highly expressed in both the HLCZ01 and HepG2-NTCP cell lines (Fig. 1C). The mRNA levels of the hepatocyte markers AAT, ALB, HNF4, and CYP3A4 in HLCZ01 cells were similar to those in HepG2-NTCP cells (Fig. 1D). Compared with HepG2-NTCP cells, HLCZ01 proliferation was significantly slower (Fig. 1E). Overall, HLCZ01 cells have typical hepatocyte characteristics and are highly differentiated hepatocellular carcinoma cells that are morphologically closer to primary human hepatocytes.

### Optimization of HBV infection efficiency

**Fig 2:**
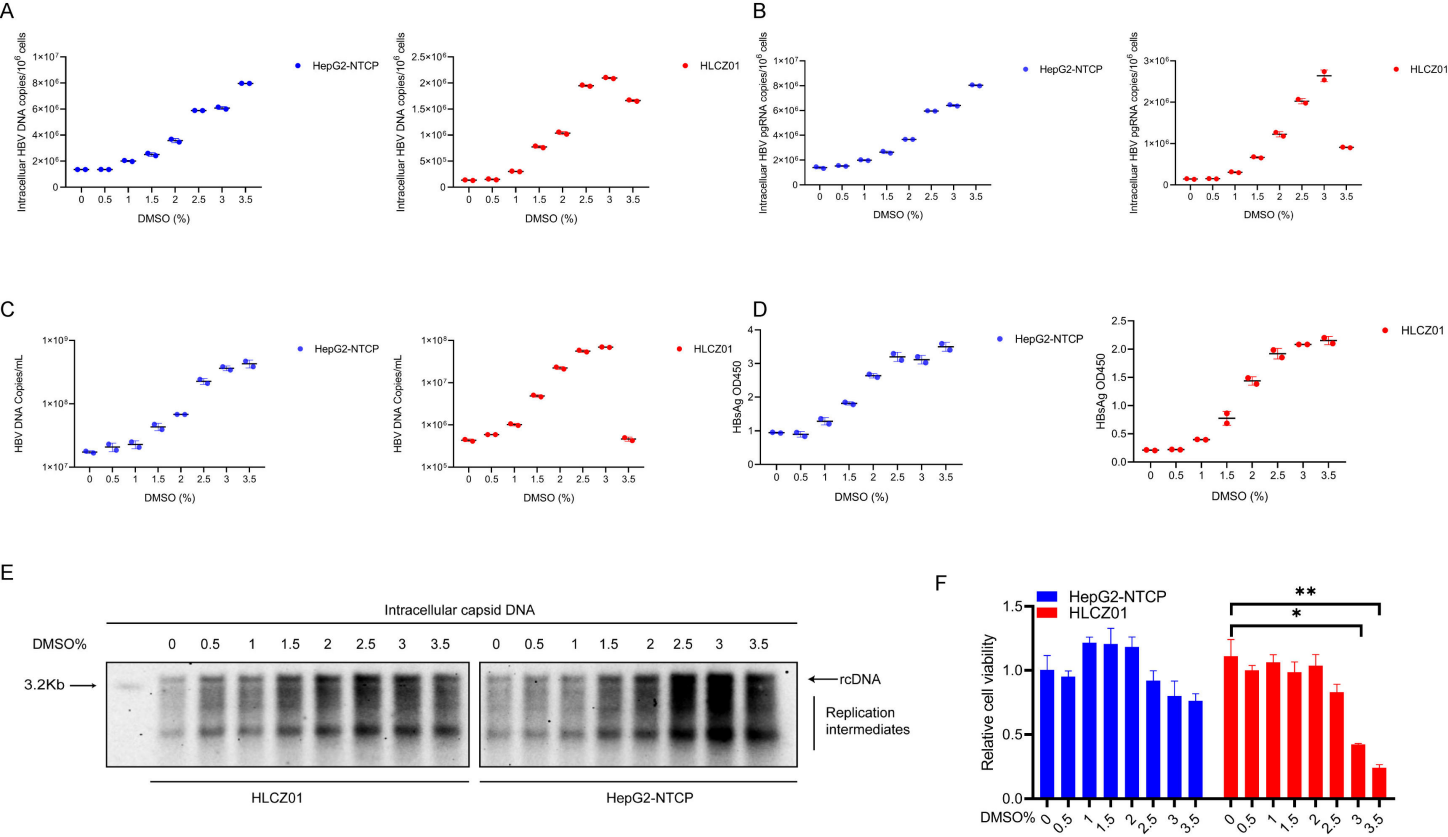
Optimization of HBV infection efficiency in HLCZ01 and HepG2-NTCP cells. (**A–E**) HLCZ01 and HepG2-NTCP cells were cultured with increasing concentrations of DMSO (up to 3.5%) 1 day after HBV infection. At 7 dpi, (**A**) intracellular HBV DNA measured by qPCR is shown as the number of HBV DNA copies per 10^6^ cells. (**B**) The viral pgRNA level determined by real-time RT-PCR is shown as the number of HBV pgRNA copies per 10^6^ cells. (**C**) The viral DNA in the supernatant, as determined by qPCR, is shown as the number of HBV copies per milliliter of supernatant. (**D**) HBsAg in the supernatant of HBV-infected cells was detected by ELISA. (**E**) Intracellular capsid-associated DNA was extracted and subjected to Southern-blot analysis using an HBV-DNA probe. (**F**) HLCZ01 and HepG2-NTCP cells (1 × 10^3^ cells/well) were seeded in 96-well plates. After 24 h, the cells were stimulated with DMSO for 48 h, and then, the cell proliferation activity was measured by CCK-8 assay. Student’s *t*-test (*P < 0.05, **P < 0.01, and ***P < 0.001 vs control).

Dimethyl sulfoxide (DMSO) maintains the functional integrity of nonproliferating mammalian hepatocytes (including human hepatocytes) and ensures their differentiation status, and the addition of certain concentrations of DMSO to the culture medium can significantly promote the HBV infection process (16). Therefore, based on a cellular model constructed in our laboratory, we explored the effect of different concentrations of DMSO on HBV replication. HBV markers, including intracellular viral DNA (Fig. 2A) and pgRNA (Fig. 2B), as well as HBV DNA (Fig. 2C) and HBV S antigen (Fig. 2D) from cell supernatant, were enhanced by exposing the cells to increasing DMSO concentrations. Southern-blot (Fig. 2E) assays further verified the above results. The HLCZ01 cells reached the highest infection efficiency at a DMSO concentration of 2.5%, after which the infection efficiency showed a decreasing trend, probably because the DMSO concentration reached a level that affected the cellular activity of HLCZ01 cells (Fig. 2F). With increasing DMSO concentration, HBV replication in HepG2-NTCP cells was enhanced in a comparable manner. To maintain the replication effect of HBV under the condition of cell viability, we chose to culture infected cells at a concentration of 2.5% DMSO.

### HBV infection kinetics in HLCZ01 and HepG2-NTCP cells

**Fig 3:**
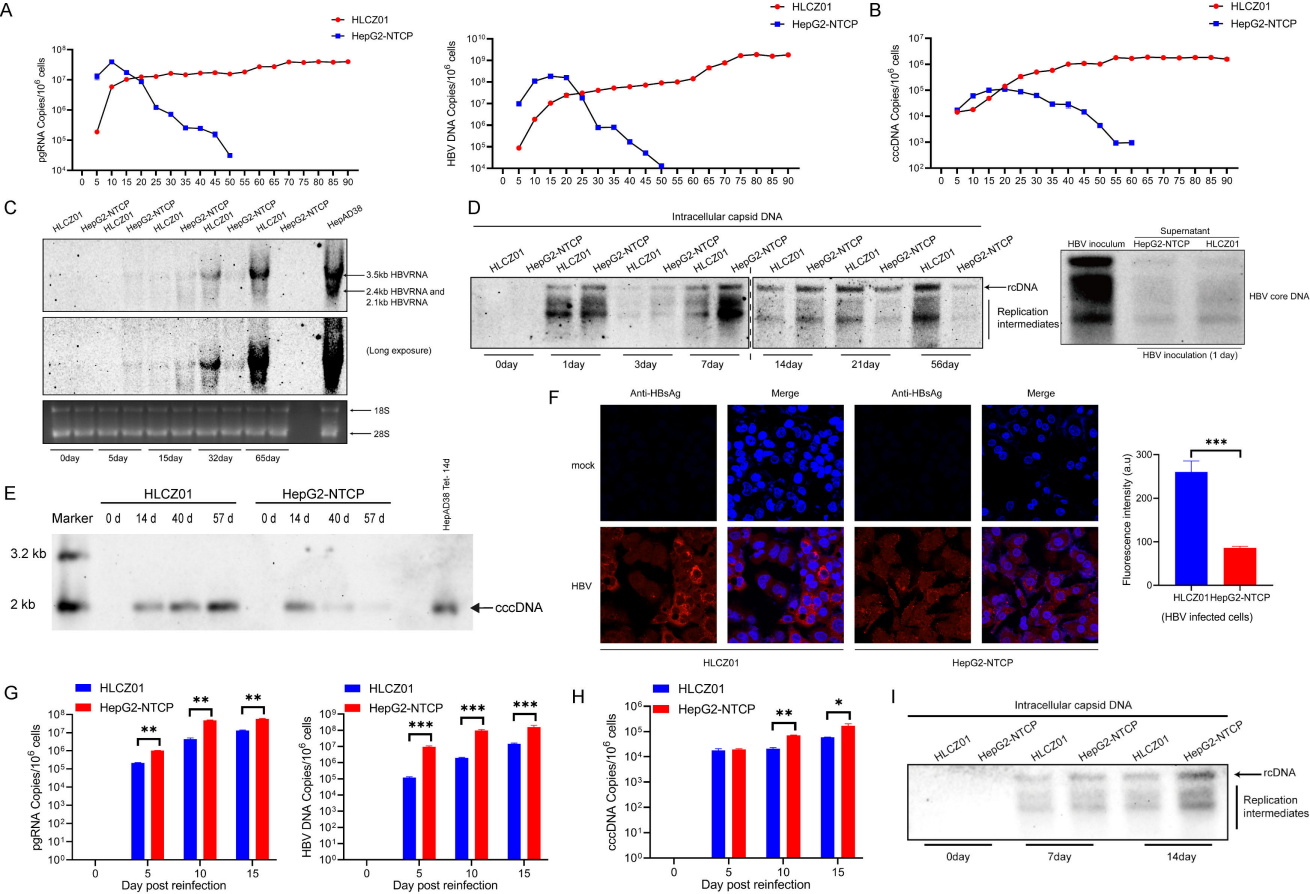
HBV infection kinetics in HLCZ01 and HepG2-NTCP cells. (**A–E**) HLCZ01 and HepG2-NTCP cells were infected with HBV at an MOI of 200 vp/cell. At the indicated time points, (**A**) viral pgRNA (left) and intracellular HBV DNA (right) measured by real-time RT-PCR or qPCR are shown as the number of HBV pgRNA or DNA copies per 10^6^ cells. (**B**) HBV cccDNA measured by qPCR with Taq DNA polymerase is shown as the number of cccDNA copies per 10^6^ cells. (**C**) Total RNA, (**D**) intracellular capsid-associated DNA, extracellular viral DNA, and (**E**) cccDNA were detected by northern- or Southern-blot analysis, respectively, using an HBV-DNA probe. (**F**) Immunofluorescence of HBsAg in HBV-infected cells. HLCZ01 and HepG2-NTCP cells were inoculated with HBV at an MOI of 200 vp/cell. Cells were harvested for immunostaining at 21 dpi using mouse monoclonal anti-HBsAg antibody. DAPI was used for nuclei counterstaining. Identical settings were maintained for image capture. Detection of viral DNA (**G**) and cccDNA (**H**) in HLCZ01 and HepG2-NTCP cells inoculated with the supernatant of HBV-infected HLCZ01 cells. (**I**) Southern blot for intracellular capsid-associated HBV DNA. HLCZ01 and HepG2-NTCP cells were treated as described in G. Student’s *t*-test (*P < 0.05, **P < 0.01, and ***P < 0.001 vs control).

To compare the infection efficiency of HBV in different cellular models, we performed long-term infection for dynamic observation (>3 months). By quantifying pgRNA, HBV DNA, and cccDNA, HBV was found to have more efficient replication in HepG2-NTCP cells in the short term, and pgRNA, HBV DNA, and cccDNA rose more rapidly in HepG2-NTCP cells than in HLCZ01 cells (Fig. 3A and B). After 3 weeks, the viral infection gradually decreased and could not sustain a prolonged infection process (Fig. 3A and B). Northern-blot (Fig. 3C) or Southern-blot (Fig. 3D and E) assays further verified the above results. Notably, HLCZ01 cells inoculated with HBV showed a trend of steadily increasing HBV DNA and cccDNA content as the duration of infection increased, whereas HepG2-NTCP cells did not seem to be capable of maintaining the cccDNA pool for a long time (Fig. 3B and E). Immunofluorescence assays also confirmed that HLCZ01 cells showed infection, and viral replication remained strong under long-term infection conditions (Fig. 3F). To investigate whether the virus particles extracted from HBV-infected HLCZ01 cells could infect naive HLCZ01 and HepG2-NTCP cells, we collected HBV-infected HLCZ01 cell supernatants and inoculated naive HLCZ01 and HepG2-NTCP cells. Viral DNA (Fig. 3G and I) and cccDNA (Fig. 3H) were detected when naive cells were inoculated with HBV-infected HLCZ01 cell supernatant. These data demonstrate that HLCZ01 supports long-term stable infection with HBV.

### Mechanism of long-term HBV infection in HLCZ01 cells

**Fig 4:**
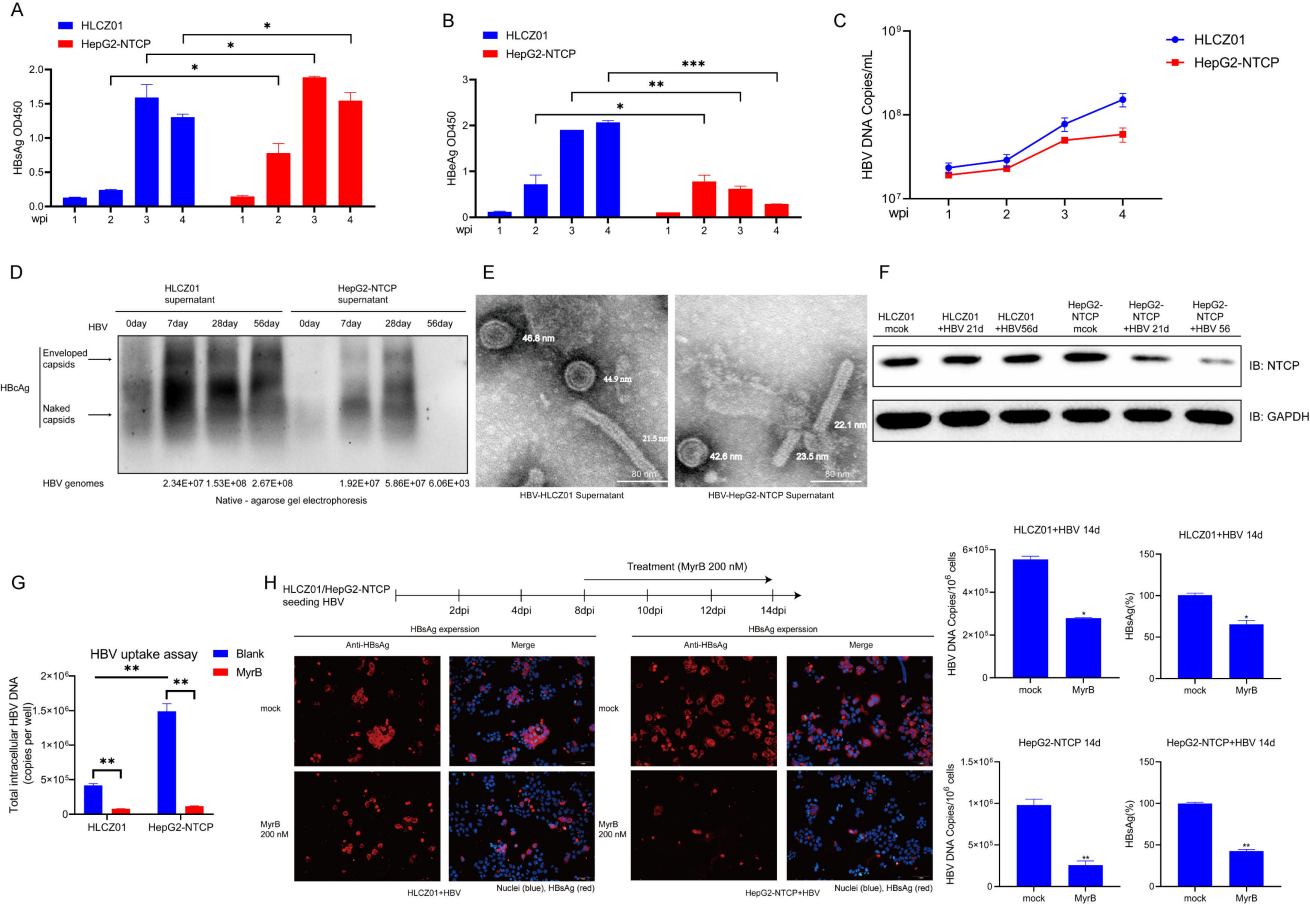
Long-term HBV infection in HLCZ01 cells. (**A–C**) HLCZ01 and HepG2-NTCP cells were infected with HBV at an MOI of 200 vp/cell. At the indicated time points (wpi represents weeks), HBsAg (**A**) and HBeAg (**B**) in the supernatant of HBV-infected cells were detected by ELISA. (**C**) The viral DNA in the supernatant, as determined by qPCR, was shown as the number of HBV copies per milliliter of supernatant. (**D**) HBcAg of enveloped capsids (virions) and nonenveloped naked capsids were analyzed by native western blotting. (**E**) Negative-staining electron microscopy of HBV particles produced by infected cells. (**F**) Western blot detection of NTCP expression in HBV-infected cells at the indicated time points. (**G**) HBV uptake was determined in the presence or absence of the entry inhibitor MyrB (200 nM). Quantification of intracellular HBV genome by qPCR. (**H**) HLCZ01 and HepG2-NTCP cells were infected with HBV and maintained with or without MyrB (200 nM) as prompted from the time of infection. HBsAg levels in HBV-infected cells were detected using immunofluorescence. Viral DNA and HBsAg levels were detected by qPCR or ELISA, respectively. Student’s *t*-test (*P < 0.05, **P < 0.01, and ***P < 0.001 vs control).

**Fig 5:**
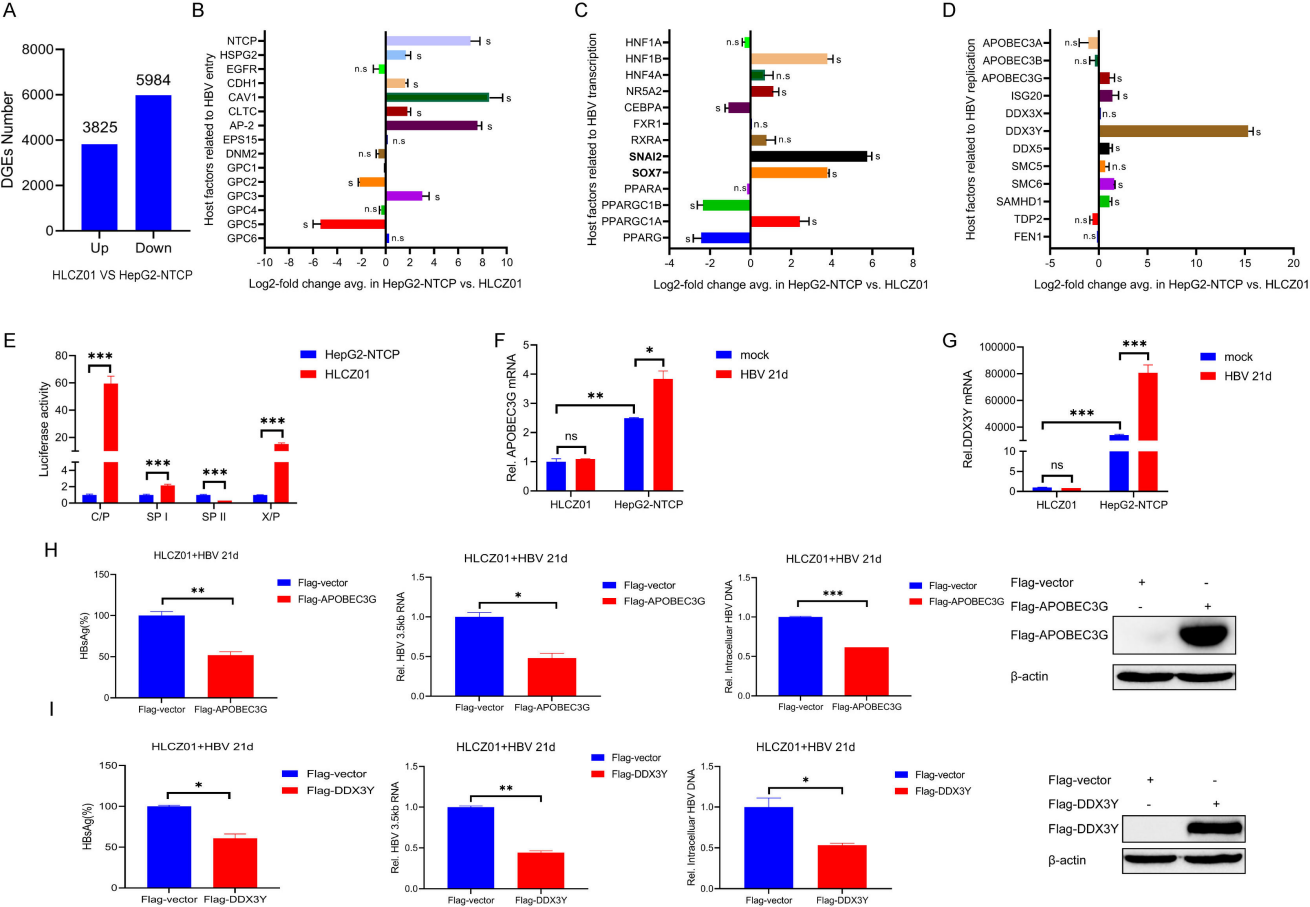
Mechanism of long-term HBV infection in HLCZ01 cells. Total RNA was isolated from HLCZ01 and HepG2-NTCP cells. A cDNA library was generated and sequenced from mRNA. (**A**) Bar graph of differentially expressed genes in HepG2-NTCP cells relative to HLCZ01 cells comprising a total of 9,809 genes. (**B–D**) Log2-fold change in gene expression related to HBV entry (**B**), transcription (**C**), and regulation (**D**) for HepG2-NTCP cells relative to HLCZ01 cells. (**E**) Plasmids containing the HBV promoter (C/P, SPI, SPII, and X/*P*) were transferred into HepG2-NTCP or HLCZ01 cells, and a luciferase reporter gene assay was used to detect the activation of the promoter. (**F–G**) HLCZ01 and HepG2-NTCP cells were infected with HBV for 21 days with an MOI of 200 vp/cell. A3G (**F**) and DDX3Y (**G**) mRNA were detected by real-time RT-PCR and normalized to GAPDH. (**H–I**) HLCZ01 cells were infected with HBV for 21 days with an MOI of 200 vp/cell and then transduced with lentivirus expressing Flag-vector, Flag-A3G, and Flag-DDX3Y, respectively. Western-blotting analysis for A3G (H Right) and DDX3Y (I Right) in HBV-infected HLCZ01 4 days after lentiviral transduction. Viral pgRNA and intracellular HBV DNA measured by real-time RT-PCR or qPCR are shown as the number of HBV pgRNA or DNA copies per 10^6^ cells. HBsAg in the supernatant of HBV-infected cells was detected by ELISA. The n.s. and s in this figure represent nonsignificant differences (n.s.) and significant differences (S) for this gene, and this significance statistic is derived from the transcriptome difference analysis with a binding threshold of fold change >2, Padj <0.05.

It was discovered through prolonged kinetic observations that HLCZ01 cells can be infected by HBV for a long period of time, while in HepG2-NTCP cells, the virus decreases rapidly after a short period of time. There are two possible explanations for this experimental phenomenon: differences in the composition of viral particles released after infection in these two cell lines or differences in the replication efficiency of HBV in the cells. Less HBV S antigen was released from the supernatant of HBV-infected HLCZ01 cells compared to that of HepG2-NTCP cells (Fig. 4A), but among the offspring virus released by HLCZ01 cells infected with HBV, relatively more were intact viral particles (Fig. 4C through E). Interestingly, HLCZ01 cells released more HBV E antigen after HBV infection than HepG2-NTCP cells (Fig. 4B). NTCP serves as a key receptor for HBV invasion. We compared the changes of NTCP expression in two cell lines (HLCZ01 and HepG2-NTCP) under HBV infection and found that HLCZ01 cells infected with HBV for a short or long time had comparable levels of intracellular NTCP protein compared with the original HLCZ01 cells, whereas NTCP expression in HepG2-NTCP cells gradually declined with the duration of infection (Fig. 4F). With the help of HBV uptake assay, it was found that the viral uptake ability of HepG2-NTCP cells was stronger than that of HLCZ01 cells after the addition of virus at the initial time point, but the process was still blocked by MyrB (NTCP inhibitor), demonstrating NTCP dependence (Fig. 4G). Although NTCP expression remained stable during HBV infection of HLCZ01, it was unknown whether HLCZ01 cells infected with HBV depended on NTCP. The addition of MyrB (NTCP inhibitor) to HLCZ01 cells infected with HBV resulted in a significant decrease in HBV infection compared to the control group, as evidenced by a significant decrease in both HBsAg and HBV DNA indicators after the addition of the inhibitor (Fig. 4H). These results indicate that HLCZ01 cells infected with HBV depended on NTCP receptors. To further investigate the factors responsible for the differential replication of HBV in the two cell lines, we performed transcriptome sequencing analysis to compare the total transcriptome consisting of 38,746 genes, with 9,809 genes significantly differentially expressed in HepG2-NTCP and HLCZ01 (3,825 genes upregulated and 5,984 genes down-regulated in HLCZ01) (Fig. 5A). HBV invasion into hepatocytes involves two steps (17): (i) HBV binds to HSPG2 with low affinity and viral receptor NTCP with high affinity to attach to the hepatocyte surface, separately; (ii) HBV enters intracellularly through endocytosis. As shown by sequencing analysis, the mRNAs of HSPG2 and NTCP in HLCZ01 cells were lower than those in HepG2-NTCP cells, and the relevant host proteins involved in viral internalization such as CDH1, CAV1, CLTC, and AP-2 were also lower than those in HepG2-NTCP cells, suggesting that HepG2-NTCP cells may be more favorable for HBV virus uptake (Fig. 5B). We confirmed this conclusion by *in vitro* uptake experiments (Fig. 4G). Compared to HLCZ01 cells, HepG2-NTCP cells expressed higher levels of genes associated with HBV transcriptional repression (Fig. 5C) (18), including SNAI2 and SOX7, key host factors known to inhibit HBV core promoter activation, which leads us to speculate that the microenvironment of HLCZ01 cells may be more favorable for HBV genome transcription, and we verified this hypothesis by performing a luciferase reporter gene assay *in vitro*. Compared with HepG2-NTCP cells, HLCZ01 cells are more conducive to HBV promoter activation (Fig. 5E). In addition, the expression of some host factors known to inhibit HBV replication (Fig. 5D), such as DDX3Y and A3G, was lower in HLCZ01 cells than in HepG2-NTCP cells, suggesting that HLCZ01 cells may be more conducive to long-term stable HBV infection. To further verify this idea, we examined the expression of related genes in HBV-infected cells after 21 days and found that HBV infection promoted the expression of A3G and DDX3Y in HepG2-NTCP cells (Fig. 5F and G). In addition, overexpression of A3G and DDX3Y in HLCZ01 cells could inhibit the replication of HBV (Fig. 5H and I).

### Optimization and establishment of alternative human liver chimeric mouse models using HLCZ01 cells

**Fig 6:**
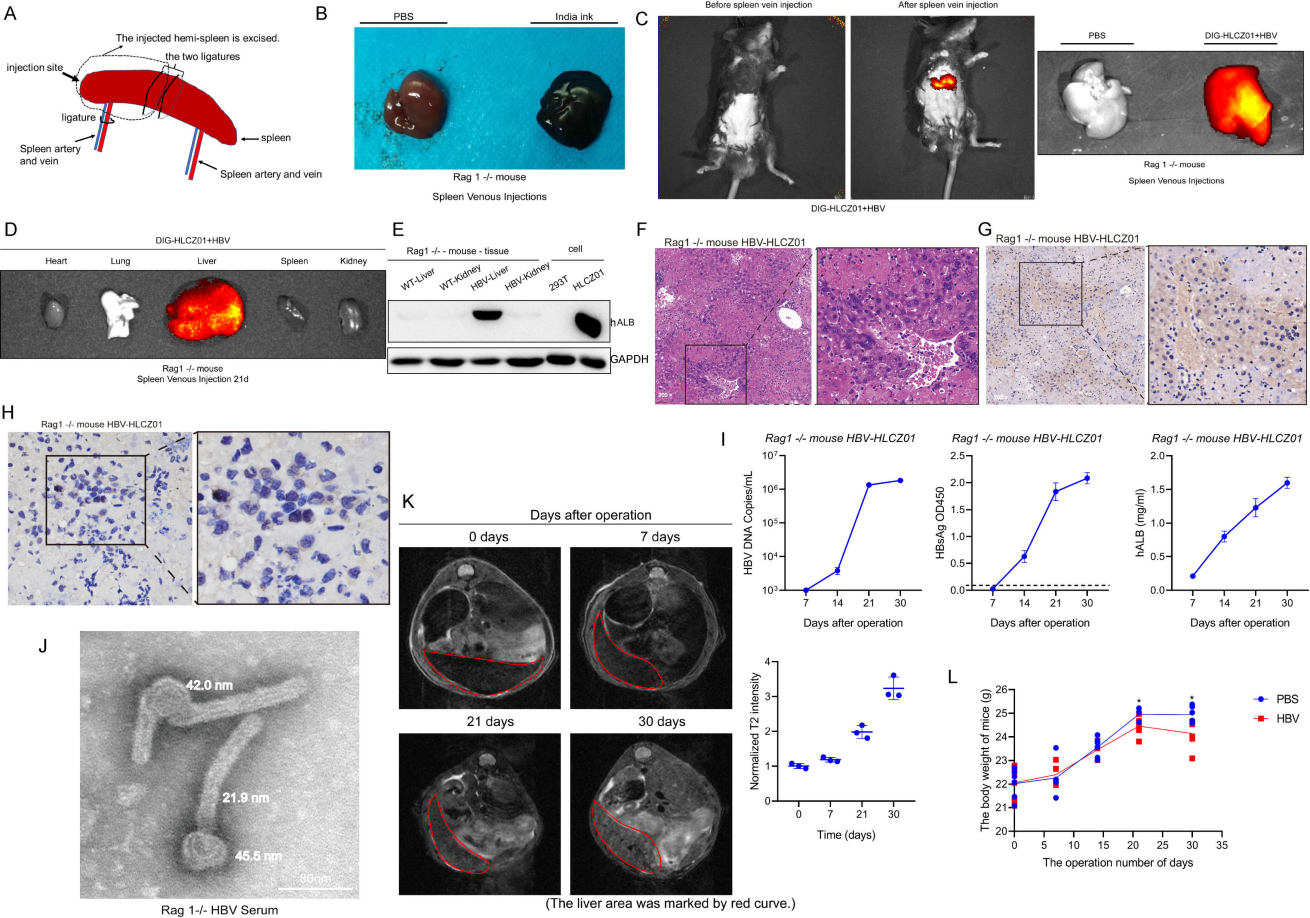
Optimization and establishment of alternative human liver chimeric mouse models using HLCZ01 cells. (**A**) Schematic diagram of the surgical approach to splitting the spleen. (**B**) After the administration of India ink through the spleen, the liver absorbs the ink. (**C–D**) Fluorescence in the hepatic region of Rag1−/− mice after splenic tip injection of HBV-infected HLCZ01 cells stained using DIR dye. Photographs of the liver 10 min (**C**) and 21 days (**D**) after injection of cells via the splenic tip. (**E**) The expression of human-derived ALB in various tissues of mice was detected by western blot, with human liver cancer cell lines as controls. (**F–G**) Representative thin section images of HBV-infected HLCZ01 cells in a Rag1−/− mice liver at 30 days post-injection of 1 × 10^6^ cells via the splenic tip. Livers were formalin-fixed, paraffin-embedded, sectioned, and H&E staining (**F**) or stained with anti-human-derived ALB (**G**) and anti-HBV core antibody (**H**) for HBV detection. (**I**) At the indicated time points (wpi represents weeks), HBsAg and human-derived ALB expression in mouse serum were detected by ELISA. The viral DNA in mouse serum, as determined by qPCR, is shown as the number of HBV copies per milliliter of supernatant. (**J**) Negative electron microscopic staining of the serum of chimeric mice producing HBV particles. (**K**) T2-MRI images of mice infected HBV-infected HLCZ01 cells (left). Normalized T2-MRI intensity (right). (**L**) The body weight changes in mice injected with phosphate buffered saline (PBS) or HBV-HLCZ01 cells after surgical manipulation.

The lack of suitable animal models has restricted studies of HBV. The most commonly used model is the human liver chimeric mouse model triggered by urokinase, but its large-scale use is hampered by its cumbersome operation, restricted access to tissue sources of primary human hepatocytes, complex genetic background, and harsh culture conditions. To explore the possibility of using HLCZ01 cells for the construction of HBV animal models, we attempted to introduce HBV-infected HLCZ01 cells into the liver by spleen injection with the help of a human liver chimeric mouse model (Fig. 6A). The accuracy of the spleen injection site can be confirmed by using India ink dye for the injection. Spleen injection causes immediate delivery of the ink specifically to the liver (Fig. 6B). HBV-infected HLCZ01 cells were stained using 1,1’-dioctadecyl-3,3,3’,3’-tetramethylindotricarbocyanine iodide (DIR) dye and subsequently injected into the liver via the hemisection spleen method (Fig. 6C). With the help of small animal imaging techniques, HBV-infected HLCZ01 cells were found to persist in the liver sites of mice (Fig. 6D). Human albumin was highly expressed in the livers of chimeric mice compared to control mice (Fig. 6E). H&E staining showed the presence of heterogeneous structures within the livers of human liver chimeric mice (Fig. 6F). Diffuse expression of Human albumin (Fig. 6G) and HBcAg (Fig. 6H) proteins located in the cytoplasm and nucleus was found in the livers of human liver chimeric mice. By collecting serum at different time points for testing HBV markers (Fig. 6I), HBV S antigen, and HBV DNA in serum, it was found that viral markers gradually accumulated in mouse serum over time, and human-derived albumin was also detected in mouse serum, indicating that persistent HBV-infected hepatocytes had chimerized into the mice. Negative-stain electron microscopy also confirmed the presence of intact viral particles in the serum of mice (Fig. 6J). Notably, magnetic resonance imaging (MRI) experiments demonstrate the absence of obvious tumor nodules in the mouse liver, but the T2 signal shows some degree of enhancement (Fig. 6K). Mice injected with HBV-HLCZ01 cells showed a slight decrease in body weight over time compared to controls (Fig. 6L). The analysis in combination with H&E staining experiments showed that there was some tumor heterogeneity in the mouse liver. This may be explained by the low malignancy of HLCZ01 cells, a highly differentiated hepatocellular carcinoma cell type with low tumorigenicity.

## DISCUSSION

The study of HBV is limited by the lack of cell culture models. Here, we constructed a hepatocellular carcinoma cell line named HLCZ01 that can maintain long-term HBV infection. Compared with the existing commonly used hepatocellular carcinoma cell line, HepG2-NTCP, we found by detecting viral pgRNA, HBV DNA, etc. that the HLCZ01 cell line can maintain HBV viral genome accumulation for a long time without significant loss. After prolonged infection, the production of offspring viruses also increased in HLCZ01 cells. In contrast, HepG2-NTCP cells were efficiently infected for a short period of time but did not maintain stable virus infection for a long period of time. Because of their morphological fullness and slow growth, HLCZ01 cells can replace primary hepatocytes as an optimal model of HBV infection. HBV-infected HLCZ01 cells can be transplanted into the livers of immunodeficient mice by borrowing the method of human liver chimeric mouse transplantation and exist in mice for a long period of time to release virus particles, which can be employed to construct an animal model of viral infection. HBV infection shows a focal pattern, indicating that HBV virus propagation may occur through a cell-to-cell attachment process (Fig. 3F and 4H). In contrast, the infection process of HepG2-NTCP cells does not show such a phenomenon. Combined with the clinical observations, HLCZ01 cells are a more consistent model of the infection process in the physiological state.

To investigate the specific mechanism by which HLCZ01 cells maintain stable HBV infection for a longer period of time compared to HepG2-NTCP cells, we compared the products released from these two cell lines following HBV infection and found that the proportion of intact virus particles released by HLCZ01 cells is higher than that released by HepG2-NTCP cells. Our speculation was further verified by nondenaturing gel experiments. To explore why HLCZ01 maintains stable HBV infection for a long period of time, we further sequenced the transcriptome of HLCZ01 cells and compared it with that of HepG2-NTCP cells. Many genes associated with HBV invasion, such as NTCP, HSPG2, CDH1, CAV1, CLTC, and AP-2, were highly expressed in the HepG2-NTCP cells obtained from the screen, which explains why the HepG2-NTCP cell line supports acute HBV infection. Interestingly, the suppressor genes associated with HBV transcription (Slug, SOX7, and DDX3Y), retrotransposition (A3G), and cccDNA stabilization (ISG20) were also highly expressed in HepG2-NTCP cells. This suggests that compared to HLCZ01 cells, HBV was blocked in intracellular circulation in HepG2-NTCP cells, making it more difficult to maintain the cccDNA pool (Fig. 3B and E) and therefore unable to undergo long-term stable infection.

HBV is considered to be a “stealth” virus that does not induce an obvious immune response during infection but can affect the immune microenvironment of the liver by releasing subviral particles and altering the metabolic reprogramming of host cells (19 – 21). Hence, the regulatory network between the virus and the immune system and the effect on the biological function of host cells can be explored based on the property that HLCZ01 cells can be stably infected by HBV for a long period of time (14, 22). It has become an academic fact that chronic HBV infection tends to develop into cirrhosis and hepatocellular carcinoma, but the mechanism of hepatocarcinogenesis remains to be elucidated because the process of viral carcinogenesis takes a long time and is influenced by numerous factors (23). The effect of HBV on cell proliferation as well as carcinogenicity can be appropriately explored by comparing HepG2 cells with HepG2.2.15 cells, but because the HepG2.2.15 cell line was constructed decades ago through resistance screening and because of subtle differences in culture conditions in numerous laboratories, HepG2.2.15 cells have become phenotypically different from their source cell line, HepG2 cells. Therefore, these two cell lines can no longer form a good control relationship with each other, and the effect of HBV in terms of cancer promotion cannot be explored in depth using these two cell lines (9). Although HepG2 cells stably overexpressing NTCP can be infected by HBV and support a complete HBV life cycle, they are not suitable for studies related to HBV carcinogenesis because of their genetical modifications, and the ability of HBV to persist in them for a long period of time remains controversial (24). In contrast, HBV can stably infect the HLCZ01 cell line for a long period of time without significant loss of its genome, so it is a good model for studying HBV-associated liver cancer.

Mice do not show susceptibility to HBV under natural conditions, so many topics have been addressed to modify mouse models at the tissue and genetic levels to support hepatitis virus infection (6, 25). One of the most common approaches is to construct human liver chimeric mice by implanting primary human hepatocytes into mouse livers to support HBV infection, but this process is not well characterized for reproducibility because of the long cycle time, variable cell sources, and unstable genetic background (11). Our data suggest that HBV is capable of long-term stable infection in the untransformed hepatocellular carcinoma cell line HLCZ01, so we attempted to use it as a substitute for primary human hepatocytes in the construction of human liver chimeric mice. We detected the viral antigen and genome of HBV, as well as human-derived albumin, in mouse serum. The presence of human albumin in the blood of mice was also found by immunoblotting analysis. The presence of viral particles in mouse serum was confirmed by electron microscopy. The presence of the HBV virus in mouse liver was verified. HLCZ01 is a stable highly differentiated hepatocellular carcinoma cell line that can be cultured for a long period of time by passaging, which has the advantages of excellent operability and a stable genetic background. In comparison with the commonly used genetically modified cell line HepG2-NTCP, HLCZ01 cells can better simulate the natural HBV infection process and are also more conducive to the construction of mouse models (Fig. 1B through D).

Overall, we have constructed an excellent cell system for HBV infection that can support the long-term infection process of HBV and mimic chronic HBV infection, and the long-term stable existence of the HBV genome implies that it can be used as an alternative to primary human hepatocytes for the construction of mouse models for HBV infection. This platform will be beneficial for the screening of antiviral drugs and vaccine development.

## MATERIALS AND METHODS

### Cell line and clone selection

HLCZ01 cells are a hepatocellular carcinoma cell line constructed by our laboratory. The specific method of construction of HepG2-NTCP cells was described in previous literature (26). Briefly, HepG2 cells purchased from ATCC were infected with a lentivirus harboring an encoded human NTCP, and infected cells were selected with puromycin (2 µg/mL) to screen the best-growing single-cell clones for their ability to support HBV infection. HLCZ01 cells and HepG2-NTCP cells were cultured in DMEM/F12 medium containing 10% Fetal Bovine Serum (FBS), 40 ng/mL dexamethasone, and 10 ng/mL epidermal growth factor (EGF) (BD). Cell passages were inoculated onto collagen-coated plates, and passages were performed every 3–5 days under trypsin digestion.

### Production of cell culture-derived infectious HBV

Infectious HBV was derived from supernatants of HepAD38 cells. Briefly, cells were cultured utilizing DMEM medium containing 10% FBS. Starting 3 weeks after induction of HBV replication, supernatants were collected weekly at 4, 5, and 6 weeks after induction and supplemented with fresh medium. The collected supernatants were pooled and aseptically filtered through a 0.2 µm filter and then mixed with polyethylene glycol (PEG) 8,000 at a final concentration of 6%. To precipitate the virus particles, the supernatant was centrifuged at 17,600 × *g* for 60 min. The precipitate was resuspended in phosphate-buffered saline containing 10% FBS and stored at −80°C.

### HBV-infected cell culture

Cells were inoculated into 12-well plates when the cells reached approximately 80% confluence, inoculated cells with HBV (multiplicity of infection, MOI of 200 genome equivalents per cell) in the presence of 4% PEG. 20–24 h later, cells were washed and cultured in a PEG-free infection medium. The medium was changed every 2 days using an infection medium. Samples were then collected at the indicated time points for experiments. Secreted HBsAg was detected by ELISA, HBV genome was detected by quantitative qPCR, HBV particles were detected by native Western blotting, HBV relaxation loop (rc) DNA and cccDNA were detected by Southern blotting, and HBV RNA was detected by northern blot. At the indicated time points, the cells were washed with PBS and fixed in 4% paraformaldehyde, and HBV S antigen (HBsAg) was detected by immunofluorescence analysis.

### ELISA

HBsAg and HBeAg in culture supernatants were measured using commercial enzyme linked immunosorbent assay (ELISA) kits according to the manufacturer’s manuals. The human albumin assay was performed using a commercial Human Albumin ELISA Kit according to the manufacturer’s instructions.

### Real-time quantitative PCR

For RNA detection, RNA was isolated from cells using TRIzol reagent. Reverse transcription PCR of total RNA was performed using the PrimeScript RT Reagent Kit with genomic DNA (gDNA) Eraser to avoid genomic DNA contamination. qRT‒PCR was conducted using SYBR Premix Ex Taq II (Tli RNaseH Plus) or FastStart Universal SYBR Green Master Mix (ROX) with an ABI7500 thermocycler. For DNA detection, DNA was isolated from tissue samples. Real-time PCR (qPCR) for total HBV DNA was performed as described previously. Hepatic HBV DNA was measured using real-time PCR (qPCR) analysis. The primers used for PCR to detect HBV DNA were 5′-CACCTCTGCCTAATCATC-3′ (sense) and 5′-GGAAAGAAGTCAGAAGGCAA-3′ (antisense). GAPDH was used as the internal control.

### Extraction and quantification of HBV cccDNA by taq-man probe qPCR

HBV cccDNA was isolated by a modified Hirt extraction method (27). Briefly, cells in 60 mm culture dishes were lysed in 1 mL of Hirt DNA lysis solution (50 mM Tris-HCl, pH 8.0, 10 mM EDTA, 150 mM NaCl, and 0.5% SDS). Cells were lysed at 37°C for 60 min, and the cell lysate was mixed with 0.25 mL of 2.5 M KCl and incubated overnight at 4°C. The supernatant was clarified by centrifugation at 14,000 *g* for 20 min and extracted with phenol and phenol/chloroform. DNA was precipitated with ethanol and dissolved in TE buffer. The hirt extracted DNA samples were treated with 1,000 IU/ml T5 exonulease (New England Biolabs, USA) at 37°C for 1 h to remove DNA except for double-stranded closed-loop DNA. The treated hirt DNA was assayed for HBV cccDNA levels by Taq-man probe real-time PCR (qPCR). The specific primers and probes are listed in Supplementary Table.

### PEG-precipitation

Six times PEG8000 buffer (48% PEG8000 and 200 mM NaCl) was applied to the supernatant of virus-infected cells at a volume ratio of 1:5. The mixture was slowly shaken overnight at 4°C, and then centrifuged at 7,500 rpm for 30 min at 4°C. The PEG precipitate was digested with DNase I (NEB) and mung bean nuclease (NEB), and DNA extraction was performed.

### HBV core DNA extraction

The cells were lysed in lysis buffer (50 mM Tris-HCl pH 8.0, 1 mM EDTA, 0.2% NP-40, and 150 mM NaCl). After resting on ice for 15 min, the cells were centrifuged at 14,000 *g* for 15 min. The remaining cytoplasmic cell lysate was digested with DNase I at 37°C for 4 h to remove free nucleic acids. The supernatants were precipitated with PEG8000, and HBV core DNA was released by digestion with proteinase K. HBV core DNA was purified with phenol/chloroform, precipitated with ethanol, and finally dissolved in TE buffer.

### Southern-blot analysis

Briefly (28), HBV core DNA and T5 exonuclease treated hirt extracted DNA samples were separated by 1% agarose electrophoresis, transferred to nylon membranes, and exposed to molecular hybridization using the DIG kit.

### Northern-blot analysis

Total RNA was extracted by the TRIZOL extraction method and dissolved in DEPC-treated water. Samples were denatured at 68°C for 5 min, 20 µg of total cellular RNA was isolated on a 1.2% agarose gel, and northern-blot analysis was performed according to previously published procedures using the dig-labeled HBV probe described above.

### Immunofluorescence of viral protein

The cells were seeded on glass coverslips, fixed in 4% paraformaldehyde for 20 min, blocked with 1:50 goat serum for 30 min, and then incubated with mouse monoclonal anti-HBsAg (S26) antibodies (Pierce) for 1 h. The cells were washed three times with PBS and stained with fluorescence-labeled secondary antibody for 45 min. Finally, the coverslips were washed with PBS and counterstained with 4’,6-diamidino-2-phenylindole (DAPI). Fluorescence images were obtained by fluorescence microscopy.

### Electron microscopy

Viral supernatants were collected, centrifuged at 10,000 × *g* for 15 min to remove cellular debris, and concentrated 4–30 times using 100 kDa size Millipore tubes, after which all samples were loaded onto 15% and 50% sucrose cushions and centrifuged at 120,000 × *g* for 2 h. A turbid interface of two sucrose layers containing virus particles was collected for each sample, exchanged with PBS buffer (pH 7.4), and concentrated to ~500 µL using Amicon Ultra-15 tubes. Each sample was examined for the presence of virus particles under the microscope using negative staining electron microscopy.

### Nondenaturing gel

HBV particles in the culture supernatant were analyzed as previously described with minor modifications (29). Briefly, HBV particles were loaded into a 1% natural agarose gel. After electrophoresis, the pellet was transferred to a nitrocellulose membrane with TNE buffer [10 mM Tris-HCl (pH 7.4), 150 mM NaCl, and 1 mM EDTA]. The membrane was immersed in PBS containing 2.5% formaldehyde for 10 min and then fixed in 50% methanol for 30 min. After blocking with 5% BSA for 1 h, the membrane was incubated with anti-HBcAg overnight at 4°C. The secondary antibody was coupled using horseradish peroxidase. Finally, HBV particles were visualized by chemiluminescence.

### HBV uptake assay

Cells were infected with HBV (MOI of 200 genome equivalents per cell) at 4°C for 1 h. Subsequently, they were transferred to 37°C for 6 h in the presence or absence of Myrcludex B (MyrB). Cells were washed twice with PBS followed by trypsin digestion and collected. Total intracellular DNA was extracted for assay as described in the quantitative real-time PCR (qPCR) section.

### Animals and transplantation of human hepatocytes

The animal surgical protocol used was described previously with minor modifications (11, 30, 31). For model construction of liver metastasis via splenic tip injection, 6- to 8-week-old male Rag1−/− mice were purchased from Nanjing GemPharmatech Co., Ltd. Animals were housed under specific pathogen-free conditions in accordance with approved protocols and in compliance with institutional guidelines. Mice were anesthetized with oxygen-delivered 2%–2.5% isoflurane [2-chloro-2-(difluoromethoxy)−1,1,1-trifluoroethane]. Body temperature was maintained using a heating pad. Complete anesthesia was ensured by assessing the response to toe pinching and maintaining 2%–2.5% isoflurane anesthesia. After the mice were placed on their sides, a small subcostal incision was made on the skin using sterile gloves and a sterile scalpel with a sterile blade, and the incision was extended to 2–3 cm. Following this, the abdominal wall musculature was separated, and the abdominal cavity was accessed to expose the spleen, and two 6–0 wires were tied around the middle of the spleen, which was separated by two ligatures to form two halves of the spleen. A 29 G syringe was used to inject 100 µL of cells into the half spleen, and the tumor cells were allowed to drain into the liver for 10 min to provide sufficient time for most of the injected cells to reach the liver, after which the vessels under the half spleen of the injected cells were ligated, and the half spleen of the injected cells was gently removed with fine scissors, and the other half was gently placed back into the abdominal cavity. The peritoneum was closed with sterile 3–0 Vickers sutures and a tapered needle, followed by suturing of the skin. After the completion of surgery, the mice were placed on a heating pad in a clean cage without a bed for recovery. Mice were administered 0.05–0.1 mg/kg buprenorphine every 6–12 h for 72 h postoperatively for pain control. After the relatively aseptic operation, careful avoidance of blood vessels, strict hemostasis, and careful postoperative recovery, a total of 20 mice were operated, of which 19 survived, with a survival rate of 95%. All animal experiments were carried out under the supervision of the Institutional Animal Care and Use Committee of Hunan University.

### Statistical analyses

The data are presented as means ± S.D. The data were analyzed using two-tailed Student’s *t*-test. *P < 0.05, **P < 0.01, and ***P < 0.001.
